# Pre-transplant microRNA serum profiles and association with acute graft-versus-host disease in allogeneic hematopoietic stem cell transplantation

**DOI:** 10.3389/fimmu.2026.1800792

**Published:** 2026-05-28

**Authors:** Guido Smits, Silje Johansen, Kristin Paulsen Rye, Kimberley Joanne Hatfield, Franziska Görtler, Guro Kristin Melve, Håkon Reikvam

**Affiliations:** 1 K.G. Jebsen Center for Myeloid Blood Cancer, Department of Clinical Science, University of Bergen, Bergen, Norway; 2 Department of Medicine, Haraldsplass Deaconess Hospital, Bergen, Norway; 3 Department of Immunology and Transfusion Medicine, Haukeland University Hospital, Bergen, Norway; 4 Department of Clinical Science, University of Bergen, Bergen, Norway; 5 Department of Medicine, Haukeland University Hospital, Bergen, Norway

**Keywords:** acute GVHD, biomarker identification, EASIX, hematopoietic stem cell transplantation, miRNA profiling

## Abstract

**Background:**

Allogeneic hematopoietic stem cell transplantation (allo-HSCT) remains associated with significant morbidity and mortality, with acute graft-versus-host disease (aGVHD) being one of the leading contributors. Early prediction of aGVHD is essential for timely intervention, however, current diagnostic tools only detect disease after tissue damage has occurred. Circulating microRNAs (miRNAs) are potential non-invasive biomarkers for early detection or prediction of aGVHD.

**Objective:**

The objective of this study was to evaluate whether serum miRNA expression profiles obtained prior to transplantation can serve as a predictive tool for aGVHD in allo-HSCT recipients, with a focus on identifying signatures that support early detection and targeted management of post-transplant complications.

**Methods:**

We investigated the association with serum miRNA profiles and the development of early complications in 78 allo-HSCT recipients. Serum samples were collected prior to transplantation, and miRNA expression was quantified using next-generation sequencing. Statistical and bioinformatic analyses were applied to identify miRNAs associated with aGVHD (grade II–IV). Associations with the Endothelial Activation and Stress Index (EASIX) were also explored.

**Results:**

Among 78 allo-HSCT recipients, 18 patients (23%) developed aGVHD grade II–IV. Early mortality occurred in nine patients within four months post-transplantation, and seven patients relapsed within one year post-transplantation. Receiver operating characteristic (ROC) analysis identified eight pretransplant miRNAs (miR-664a-5p, miR-20b-5p, miR-93-5p, miR-25-3p, miR-1224-5p, miR-106b-5p, miR-454-3p, and miR-3679-5p) associated with subsequent aGVHD development. A predictive model based on these miRNAs achieved an AUC of 0.855, which decreased to 0.692 after bootstrap validation. Hierarchical clustering using these miRNAs separated patients into two distinct clusters with markedly different aGVHD risks: cluster 1 showed a significantly higher incidence (42% vs. 9%) and greater severity, including all grade IV cases and most miRNAs were upregulated in patients developing aGVHD. Additionally, eight other miRNAs correlated with the EASIX score, reflecting endothelial stress, although these did not overlap with aGVHD-associated miRNAs.

**Conclusion:**

Pre-transplant serum miRNA signatures can predict aGVHD risk and severity, offering a novel approach for early patient stratification. These findings support the integration of miRNA profiling into pre-transplant assessment to guide personalized GVHD prophylaxis and monitoring. Further validation in independent cohorts is warranted.

## 1 Introduction

Allogeneic hematopoietic stem cell transplantation (allo-HSCT) remains the most potent curative treatment option for many hematological malignancies and disorders (1). While allo-HSCT can provide long-term remission and potential cure, it is frequently complicated by severe immune-mediated side effects, and among them is acute graft-versus-host disease (aGVHD), in which donor-derived immune cells attack the recipient’s tissues (2). aGVHD most commonly affects the skin, liver, and gastrointestinal (GI) tract and is a major contributor to post-transplant morbidity and mortality (2). Early prediction of aGVHD is critical, as it allows timely initiation of immunosuppressive therapy, which may reduce disease severity, limit organ damage, and improve overall survival. However, current diagnostic approaches primarily rely on clinical symptoms and histopathological confirmation from biopsies, methods that identify the disease only after tissue damage has occurred (3). There is therefore a pressing need for reliable biomarkers that can predict the risk of aGVHD before clinical manifestations appear (4).

Promising candidates for such biomarkers are microRNAs (miRNAs), a class of small non-coding RNAs that regulate gene expression post-transcriptionally (5). miRNAs are stable in circulation, can be detected non-invasively in serum, and reflect immune and inflammatory processes, making them ideal for monitoring transplant-related complications (6). Several studies have identified specific miRNAs associated with the development and progression of both aGVHD and chronic GVHD (cGVHD) (4, 7). However, existing studies have primarily focused on miRNA expression after transplantation, during the onset or progression of GVHD (7–9). The predictive value of pre-transplant miRNA profiles remains largely unexplored. Investigating serum miRNA levels prior to allo-HSCT could offer a novel strategy to identify patients at increased risk of developing serious early complications, including aGVHD, before the clinical course is set in motion. Such predictive insight would support early risk stratification, personalized prophylactic strategies, and ultimately improved transplant outcomes.

The objective of this study was to determine whether serum miRNA expression profiles measured prior to transplantation can predict the development of aGVHD in allo-HSCT recipients. Specifically, we aimed to identify pre-transplant miRNA signatures associated with complication risk, to enable earlier detection and targeted interventions to mitigate post-transplant complications.

## 2 Methods

### 2.1 Patients’ characteristics

In this retrospective study, 80 consecutive allo-HSCT recipients were included. Two patients were excluded due to unavailable miRNA sequencing data, resulting in a final cohort of 78 patients. The study cohort consisted of 47 men and 31 women, with an average age of 56 years (range 17-72). The main indications for transplantation were acute myelogenous leukemia (AML) (44/78; 56%), myelodysplastic neoplasms (MDS) (17/78; 22%), and acute lymphoblastic leukemia (ALL) (11/78; 14%). None of the patients had undergone prior allogeneic transplantation. Sixty-eight patients received peripheral blood hematopoietic stem cells from a matched related donor (MRD) and 10 from a matched unrelated donor (MUD). Most patients (73/78; 94%) received cyclosporine A (CSA) and methotrexate (MTX) as GVHD prophylaxis; three received CSA and mycophenolate mofetil (MYCO), one received CSA and sirolimus (SIRO), and one received MTX and MYCO. The EASIX score was calculated by the formula (lactate dehydrogenase (LDH) x creatinine)/Platelet count (10). For analyses involving the EASIX score, one additional patient was excluded due to missing LDH values, leaving 77 patients for these specific analyses. The patient, donor and graft characteristics are given in **Table 1**.

**Table 1:** Demographical, clinical, and laboratory data for the 78 patients included in the study.

Patient and transplant characteristics	Observation	+ aGVHD	- aGVHD
**Total**	78	18	60
Demographic data and disease history			
Gender (numbers, male/female)	47/31	12/6	35/25
Age (years, median, and range)	56 (17-72)	53 (17-66)	57 (17-72)
Height (cm, median, and range)	174 (152-197)	173 (159-197)	174 (152-193)
Weight (kg, median, and range)	75 (43-139)	77 (57-110)	74 (43-139)
BMI (kg/m^2^, median, and range)	24.9 (17.3-41.4)	26.1 (19.7-31.5)	24.7 (17.3-41.4)
Diagnosis (numbers)			
AML	44	9	35
MDS	17	3	14
ALL	11	4	7
MF	2	0	2
CLL	1	0	1
HL	1	0	1
MPN	1	1	0
T-PLL	1	1	0
Conditioning regimen (numbers)			
FLU + TRE	22	4	18^a^
FLU + BU	17	7	10
BU + CY	13	5	8
FLU + TRE + ATG	12	1	11
FLU + BU + ATG	4	0	4
TBI + ETO	4	0	4
CLO + BU	1	1	0
FLAMSA + TRE	1	0	1
FLU + CY	1	0	1
FLU + CY + TBI	1	0	1
FLU + CY + TBI + ATG	1	0	1
TBI + CY	1	0	1
Laboratory data (median and range)			
Leukocytes (x10^9^/L)	3.2 (0.5-18.2)	2.4 (0.5-12.6)	3.7 (0.5-18.2)
Neutrophils (x10^9^/L)	2.1 (0.1-10.1)	1.6 (0.2-9.6)	2.1 (0.1-10.1)
Lymphocytes (×10⁹/L)	0.8 (0.2-5.7)	0.7 (0.2-2.7)	0.8 (0.2-5.7)
Monocytes (×10⁹/L)	0.5 (0-5.7)	0.4 (0.0-2.7)	0.5 (0.0-5.7)
Hemoglobin (g/dL)	9.7 (7.5-14.7)	9.8 (7.5-13.3)	9.7 (7.5-14.7)
Platelets (×10^9^/L)	168 (6-1181)	158 (17-431)	168 (6-1181)
LDH (U/L)	200 (113-810)	203 (113-344)	200 (113-810)
CRP (mg/L)	7 (1-137)	11 (1-98)	7 (1-137)
Creatinine (µmol/L)	72 (42-137)	72 (43-98)	73 (42-137)
ESR (mm/hr)	51 (3-137)	48 (5-137)	51 (3-137)
Albumin (g/L)	41 (29-49)	42 (33-46)	41 (29-49)
Protein (g/L)	67 (49-85)	69 (59-76)	67 (49-85)
Ferritin (µg/L)	1787 (45-6971)	1801 (307-3839)	1787 (45-6971)
EASIX score	99 (15-2635)	104 (37-791)	96 (15-2635)
aGVHD organ involvement (numbers)			
GI tract		13	
Skin		11	
Liver		3	
Donor/graft characteristics			
Donor gender (numbers, male/female)	51/27	10/8	41/19
Donor age (years, median, and range)	51 (18-77)	52 (23-73)	50 (18-77)
CD34^+^ cells (x 10^6^/kg, median, and range)	2.8 (6.2-10.0)	6.7 (2.8-9.5)	6.1 (3.2-10.0)
CD3^+^ cells (x 10^8^, median, and range)	236.5 (15.4-718.9)	200.8 (109.7-550.3)	255.2 (15.4-718.9)
CD3^+^ cells (x 10^8^/kg, median, and range)	3.2 (0.2-9.3)	3.3 (1.4-7.7)	3.2 (0.2-9.3)

^a^ 2 patients received FLU + TRE + post CY.

Values are given as median and range in parentheses. Height and weight were registered at the start of conditioning therapy. Abbreviations: ALL, acute lymphoblastic leukemia; AML, acute myelogenous leukemia; ATG, antithymoglobulin; BMI, body mass index; BU, busulphan; CLL, chronic lymphocytic leukemia; CLO, clofarabine; CRP, C-reactive protein; CY, cyclophosphamide; EASIX*, endothelial activation and stress index; ETO, etoposide; FLAMSA, fludarabine, cytarabine, amsacrine; FLU, fludarabine; HL, Hodgkin’s lymphoma; LDH, lactate dehydrogenase; MDS, myelodysplastic neoplasm; MF, myelofibrosis; MPN, myeloproliferative neoplasm; ESR, erythrocyte sedimentation rate; TBI, total body irradiation; T-PLL, T-cell prolymphocytic leukemia; TRE, treosulfan. * EASIX score is calculated by (LDH U/L x Creatinine (µmol/L))/Platelets × 10^9^/L.

### 2.2 Preparation of serum samples

Samples were collected during the routine clinical consultation prior to conditioning and transplantation, with a median interval of 27 days. Venous blood samples were collected in Vacuette Cat Serum Clot Activator tubes (Greiner Bio-One, Kremsmünster, Austria), allowed to coagulate for 30–120 minutes at room temperature (18 °C) and centrifuged at 2200 g for 10 minutes. Serum was then collected, immediately frozen, and stored at -80 °C until further use.

### 2.3 Diagnosis of complications

The diagnosis of aGVHD was established through comprehensive clinical evaluation in accordance with the Mount Sinai Acute GVHD International Consortium (MAGIC) criteria (11). Biopsy verification was performed to confirm the diagnosis in cases of uncertainty.

### 2.4 miRNA isolation and quantification

Total RNA was isolated from a standard volume of 200 µl serum using the miRNeasy Serum/Plasma Kit (Qiagen, Hilden, Germany) according to the manufacturer’s instructions, with an elution volume of 14 µl. Subsequently, 5 µl of total RNA was used for miRNA NGS library preparation using the QIAseq miRNA Library Kit (Qiagen) according to the manufacturer’s protocol. During the reverse transcription process, unique molecular identifiers (UMIs) were introduced to enable accurate quantification and deduplication. The libraries were amplified by PCR with index primers, purified, and their quality and concentration were assessed by capillary electrophoresis using the Fragment Analyzer (Agilent Technologies, Santa Clara, CA, USA). Libraries were pooled equimolarly based on concentration measurements and quantified by qPCR prior to sequencing on an Illumina NextSeq platform (1×75, 2×10). Demultiplexing and FASTQ file generation were performed with bcl2fastq2 (Illumina, San Diego, CA, USA).

### 2.5 Analysis of miRNAs in serum samples

Analysis was performed using CLC Genomics Server 23.0.5, using the “QIAseq miRNA Quantification” workflow with standard parameters. The common sequences, UMIs, and adapters were trimmed, and reads with a length < 15 nt or > 55 nt were discarded. The remaining reads were deduplicated using their UMIs. Reads with identical UMIs, starting positions (based on the end of the read to which the UMI was ligated), and strand were grouped. Singleton groups were merged with non-singleton groups if their UMIs could be converted by introducing a single-nucleotide polymorphism (SNP), following the default workflow. Reads were mapped to miRBase v22, allowing perfect matches and isomiRs with a maximum of 2 mismatches and/or alternative start or end positions of up to 2 nt. Reads that did not map to miRBase were mapped to the human genome (hg38) using the “RNA-Seq Analysis” workflow with default parameters. Read counts were normalized to counts per million (CPM) for downstream analyses.

### 2.6 Bioinformatical and statistical analyses

Bioinformatic and statistical analyses were performed in R (v4.5.0). Patient and donor characteristics were compared between patients who developed aGVHD (grade II–IV) and those who did not. Continuous variables were tested for normality using the Shapiro-Wilk test and compared using the independent samples t-test or Mann-Whitney U test. Categorical variables were compared using the chi-square test or Fisher’s exact test. miRNAs with a CPM value greater than 1 in at least 75 of 78 patients were selected for further analysis. Receiver operating characteristic (ROC) analyses and area under the curve (AUC) calculations were performed using the pROC package (v1.19.0.1), and miRNAs with an AUC > 0.7 were considered to have acceptable discriminatory power. An additional ROC analysis combined the selected miRNAs into a single model. Model performance was assessed using bootstrap validation with 1000 iterations using a fixed random seed to ensure reproducibility. To account for class imbalance (n = 18 aGVHD grade II-IV vs. n = 60 controls), inverse frequency class weights were applied during logistic regression model training. In each iteration, a bootstrap sample was drawn with replacement, and the model was trained on this sample with recalculated class weights. Performance was evaluated on out-of-bag samples, with the optimal classification threshold determined using the Youden index. The AUC, sensitivity, specificity, positive predictive value (PPV), and negative predictive value (NPV) are reported as means across all valid iterations.

The selected miRNAs were log10-transformed and z-score normalized. Hierarchical clustering of both miRNAs and patients was performed using Ward’s method based on pairwise Pearson correlations. The heatmap was visualized using the ComplexHeatmap package (v2.24.1). Spearman correlations between the selected miRNAs were computed and visualized using corrplot (v0.95). The correlation between miRNA expression and the EASIX score was assessed using Spearman correlation with Benjamini-Hochberg correction for multiple testing. miRNAs with an adjusted p-value < 0.05 and an absolute correlation coefficient > 0.6 were considered significantly correlated with the EASIX score. Results were visualized as scatter plots using ggplot2 (v4.0.1) and as a correlation matrix using corrplot.

## 3 Results

### 3.1 Donor or graft characteristics are not associated with aGVHD

Patient, donor, and graft characteristics were compared between patients who developed aGVHD grade II–IV (n=18) and those who did not (n=60). No statistically significant differences were observed between the two groups in any of the analyzed variables, including age, sex, diagnosis, conditioning regimen, donor type, EASIX score, CD34+ cell dose, CD3+ cell dose, or donor characteristics (all p > 0.05).

### 3.2 Incidence of early allo-HSCT complications

Following allogeneic transplantation, 18 recipients (23%) developed grade II–IV aGVHD. Disease severity was distributed as follows: one patient developed grade II aGVHD, eleven patients developed grade III aGVHD, and six patients developed grade IV aGVHD. Thus, most affected patients presented with severe disease (grade III–IV). Regarding organ involvement among patients with aGVHD, the GI tract was the most frequently affected organ (n = 13), followed by the skin (n = 10), and the liver (n = 3), with some patients presenting involvement of multiple organs (**Table 1**). Clinical outcomes in the overall cohort were also assessed. Nine patients died within four months after transplantation. Causes of death included disease relapse (n = 3), multi-organ failure (n = 3), acute GVHD (n = 2), and intracranial hemorrhage (n = 1). In addition, seven patients experienced early disease relapse within twelve months post-transplantation.

### 3.3 Presence of miRNAs in serum samples

**Figure 1:**
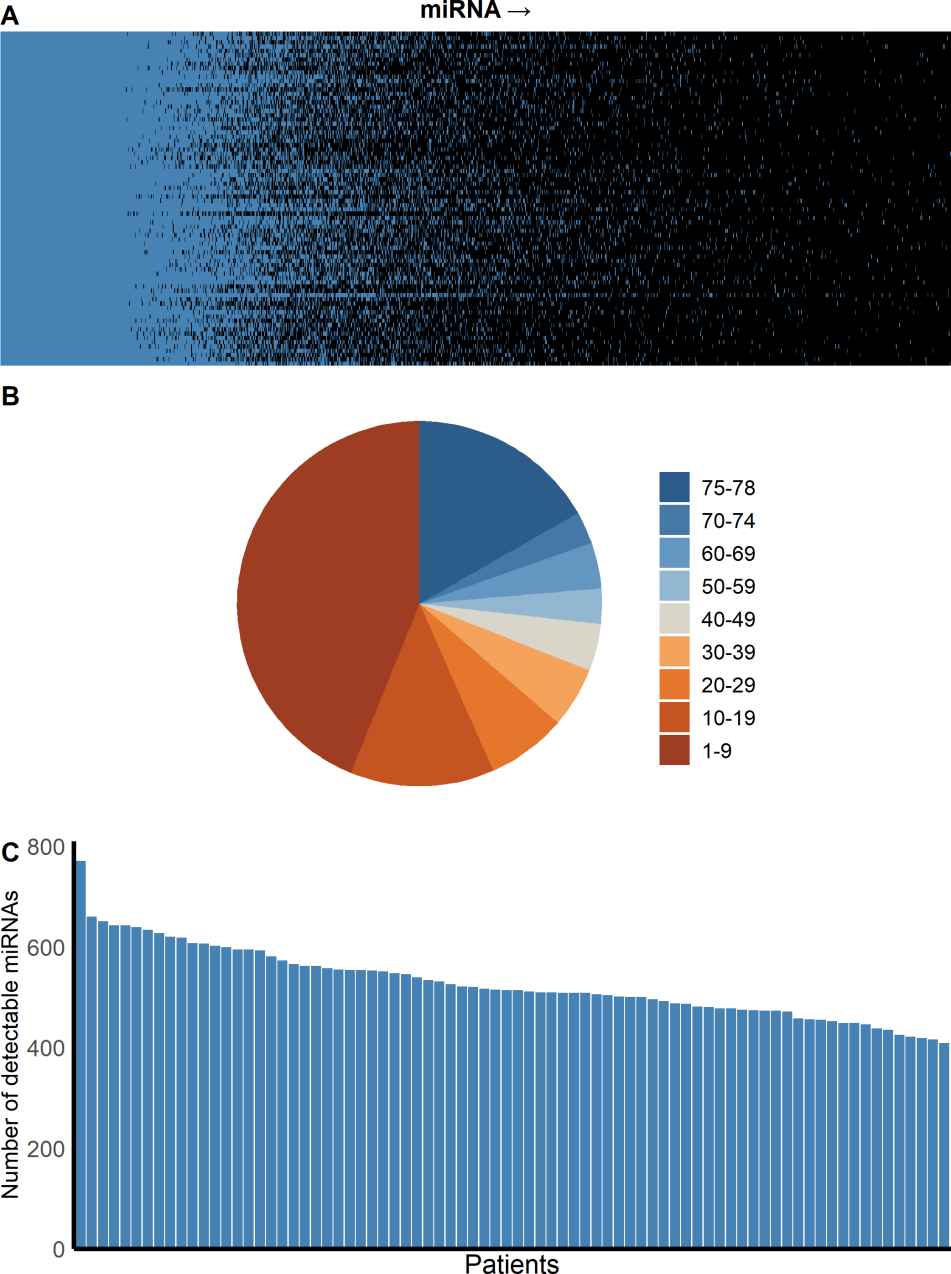
Detection of miRNAs in pre-transplant serum samples of allo-HSCT recipients. **(A)** Heatmap showing miRNA detection; blue indicates detectable levels in serum and black indicates levels below the detection limit. Patients are shown in columns and miRNAs in rows. **(B)** Distribution of detectable miRNAs among the 1,471 miRNAs identified in the study. Of these, 16.7% were detected in 75–78 patients, 2.9% in 70–74, 4.1% in 60–69, 3.1% in 50–59, 4.1% in 40–49, 5.4% in 30–39, 7.1% in 20–29, 12.8% in 10–19, and 43.8% in 1–9 patients. **(C)** Number of detectable miRNAs per individual patient, ranging from 409 to 771 (median 514).

In the study cohort, the expression levels of 2632 miRNAs were investigated. 1471 miRNAs were detected in at least one patient (**Figure 1A**). Of these miRNAs, 16.7% was detected in 75–78 patients, 2.9% in 70–74 patients, 4.1% in 60–69 patients, 3.1% in 50–59 patients, 4.1% in 40–49 patients, 5.4% in 30–39 patients, 7.1% in 20–29 patients, 12.8% in 10–19 patients and 43.8% in 1–9 patients (**Figure 1B**). Patients expressed 409–771 miRNAs, with a median of 514 miRNAs (**Figure 1C**).

### 3.4 ROC analysis identified pretransplant miRNAs associated with aGVHD

**Figure 2:**
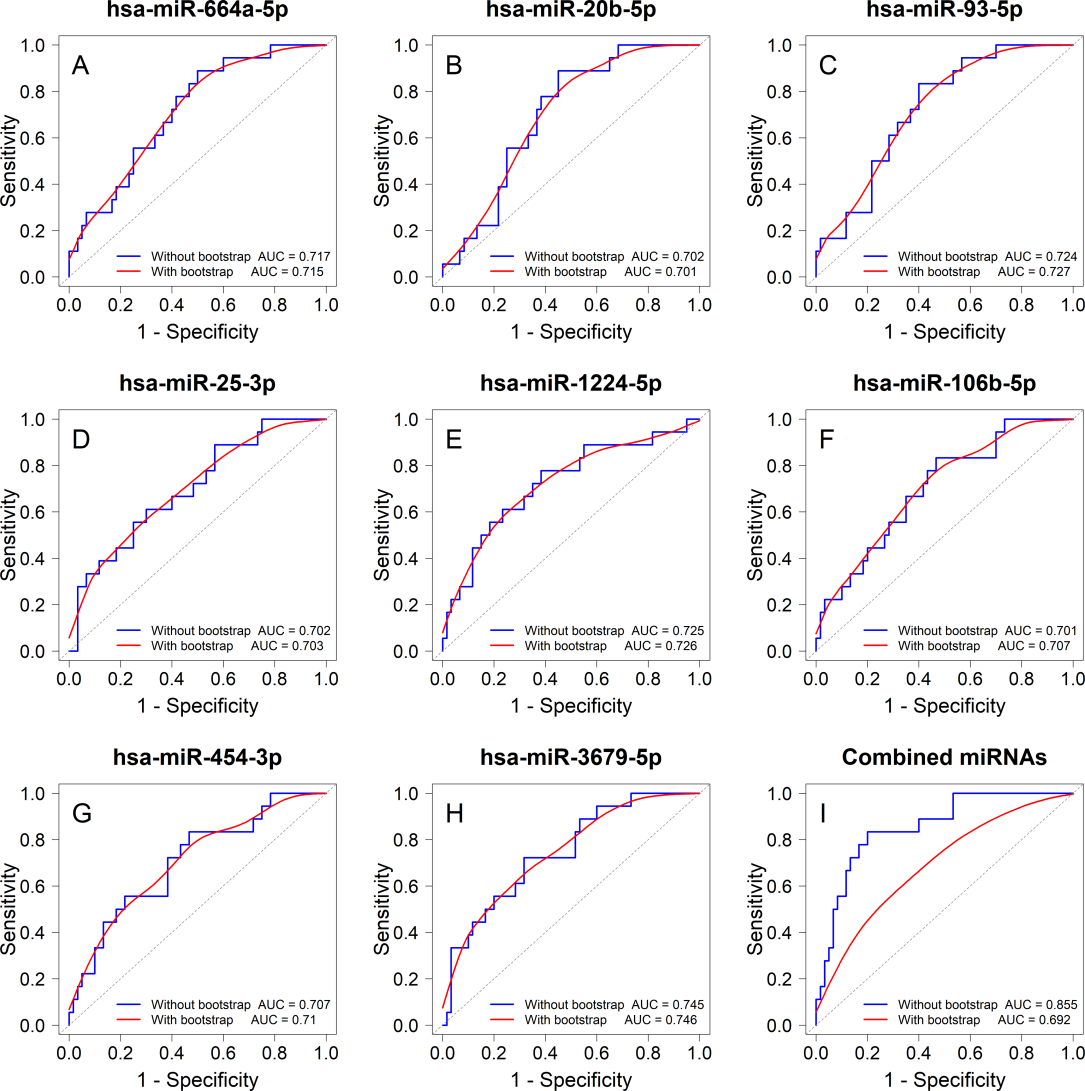
ROC curves for miRNAs associated with risk of developing aGVHD (AUC > 0.7; expressed in ≥ 75 patients). Eight miRNAs were identified: miR-664a-5p **(A)**, miR-20b-5p **(B)**, miR-93-5p **(C)**, miR-25-3p **(D)**, miR-1224-5p **(E)**, miR-106b-5p **(F)**, miR-454-3p **(G)**, and miR-3679-5p **(H)**. Panel **(I)** shows the combined model including all eight miRNAs. ROC curves are shown with and without bootstrap validation, and AUC values are indicated in the figure.

To assess the ability of each miRNA to predict the development of aGVHD grade II-IV, the AUC was calculated (**Figures 2A–H**). Eight miRNAs—miR-664a-5p, miR-20b-5p, miR-93-5p, miR-25-3p, miR-1224-5p, miR-106b-5p, miR-454-3p, and miR-3679-5p—were found to be associated with the risk of developing aGVHD. Bootstrap validation was performed on the dataset, and the positive predictive value (PPV), negative predictive value (NPV), sensitivity and specificity of the unvalidated and validated ROC curves were calculated (**Table 2**). Finally, based on the eight identified miRNAs, a linear regression model was performed to validate the predictive model. This model demonstrated the highest AUC of 0.855 without bootstrap validation and 0.692 with bootstrap validation (**Figure 2I**).

**Table 2:** miRNAs predictive for aGVHD.

		Without bootstrap validation						With bootstrap validation					
miRNA	N	AUC	Cutoff	Sens.	Spec.	PPV	NPV	AUC	Sens.	Cutoff	Spec.	PPV	NPV
hsa-miR-664a-5p	78	0.717	0.401	0.500	0.889	0.938	0.348	0.715	0.616	0.461	0.851	0.941	0.421
hsa-miR-20b-5p	78	0.702	0.419	0.550	0.889	0.943	0.372	0.701	0.593	0.434	0.866	0.942	0.392
hsa-miR-93-5p	78	0.724	0.416	0.600	0.833	0.923	0.385	0.727	0.616	0.423	0.876	0.949	0.421
hsa-miR-25-3p	78	0.702	0.384	0.433	0.889	0.929	0.32	0.703	0.660	0.478	0.760	0.911	0.465
hsa-miR-1224-5p	77	0.725	0.410	0.617	0.778	0.902	0.378	0.726	0.724	0.488	0.763	0.912	0.490
hsa-miR-106b-5p	78	0.701	0.412	0.533	0.833	0.914	0.349	0.707	0.636	0.464	0.801	0.922	0.431
hsa-miR-454-3p	78	0.707	0.440	0.533	0.833	0.914	0.349	0.710	0.678	0.481	0.766	0.908	0.457
hsa-miR-3679-5p	78	0.745	0.512	0.683	0.722	0.891	0.406	0.746	0.707	0.518	0.787	0.926	0.500
Combined miRNAs	77	0.855	0.482	0.800	0.833	0.941	0.556	0.692	0.666	NA	0.741	0.899	0.447

The table presents the values for the number of patients with detectable levels of specific miRNA (N), area under the curve (AUC), Cutoff value (Youden Index)sensitivity (Sens), specificity (Spec), positive predictive value (PPV) and negative predictive value (NPV), without and with bootstrap validation for the eight identified miRNAs with AUC > 0.7, which were expressed in >77 patients.

### 3.5 Hierarchical clustering analysis identified two distinct patient clusters

**Figure 3:**
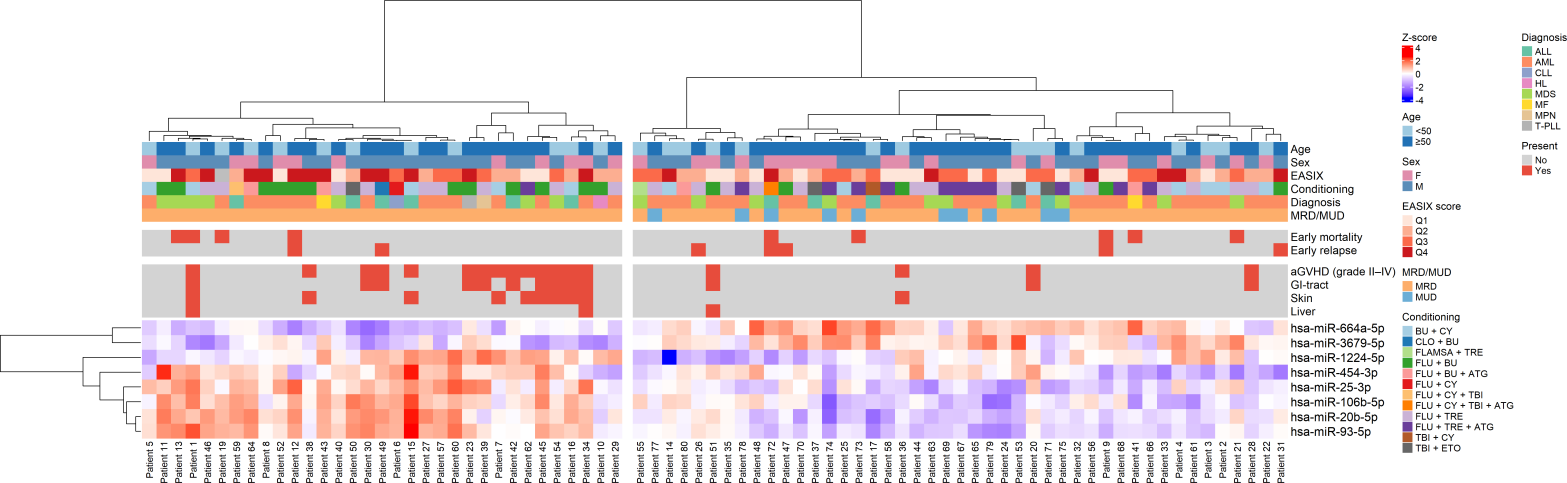
Hierarchical clustering of discrimination miRNAs. Hierarchical clustering of the eight identified miRNAs (**Figure 2**) was performed using Pearson correlation and Ward’s method. The heat map, corresponding dendrograms, and patient characteristics are shown. Red indicates high relative miRNA expression; blue indicates low expression. Patient and transplantation characteristics are displayed in the annotation tracks above. Two main patient clusters were identified; the left cluster includes most of the patients who developed aGVHD (grade II–IV), whereas the right cluster predominantly includes patients who did not develop aGVHD. Fourteen of 18 patients with aGVHD grade II-IV were identified in the first cluster, compared to 4 of 18 in the second cluster. A chi-square test demonstrated a significant difference in aGVHD occurrence between the two clusters (p = 0.001). Abbreviations: AML, acute myelogenous leukemia; MDS, myelodysplastic neoplasm; ALL, acute lymphoblastic leukemia; MF, myelofibrosis; MPN, myeloproliferative neoplasia; CLL, chronic lymphocytic leukemia; HL, Hodgkins lymphoma; T-PLL, T prolymfocytic leukemia; BU, busulphan; CY, cyclophosphamide; FLU, fludarabine; TRE, treosulfan; ATG, anti-thymoglobulin; TBI, total body irradiation; ETO, etoposide; MTX, methotrexate; SIRO, sirolimus; MYCO, mycophenolate mofetil; CSA, cyclosporine A; MRD, match related donor; MUD, match unrelated donor.

**Figure 4:**
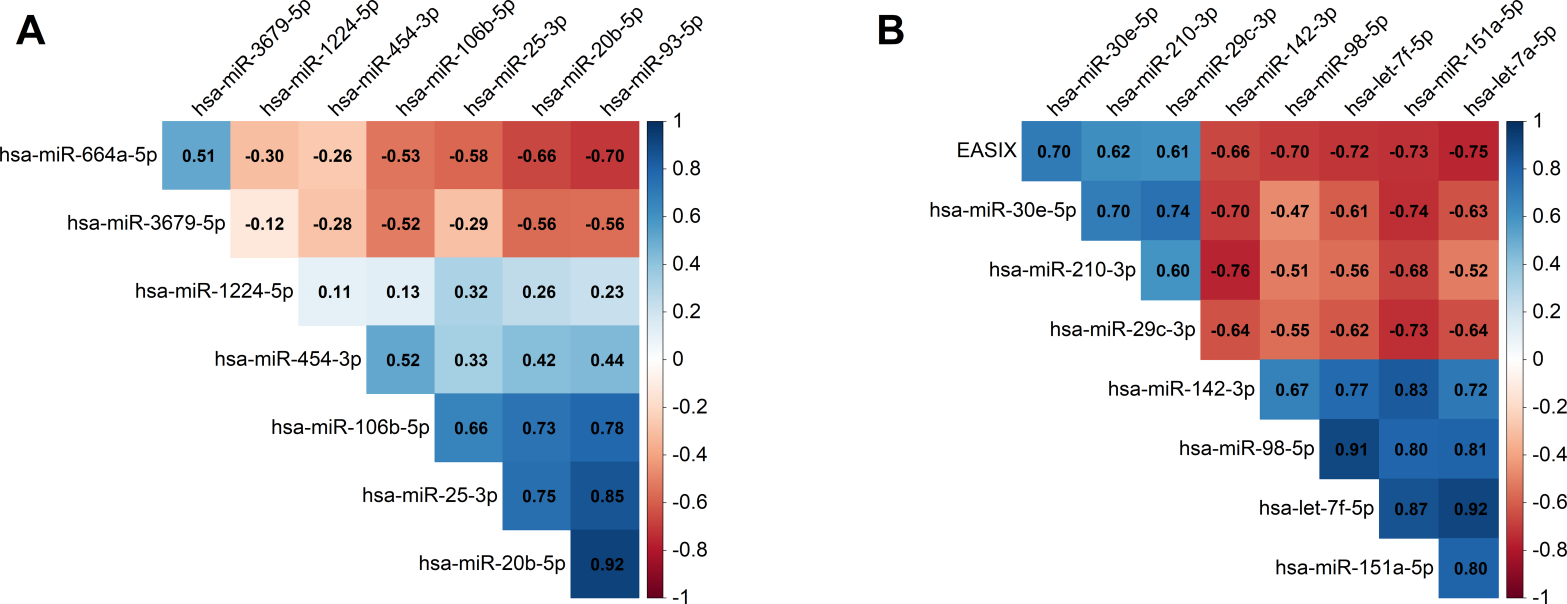
Correlation matrix. **(A)** Spearman’s correlation matrix showing a correlation between the eight highly discriminative miRNAs. Color scale ranges from -1 (dark red, strong negative correlation) to +1 (dark blue, strong positive correlation). White indicates no correlation (r = 0). The corresponding correlation coefficients are given in the squares. **(B)** Spearman’s correlation matrix showing a correlation between the eight miRNAs found to be correlated to the EASIX score. Color scale ranges from -1 (dark red, strong negative correlation) to +1 (dark blue, strong positive correlation). White indicates no correlation (r = 0). The corresponding correlation coefficients are given in the squares.

Next, a hierarchical clustering analysis was conducted based on the eight identified miRNAs. This analysis revealed two main patient clusters; cluster 1 included 33 patients, while cluster 2 comprised 45 patients (**Figure 3**). Of the 18 patients with aGVHD grade II-IV, 14 were in the first cluster, whereas only 4 were in the second cluster (**Figure 3**). The proportion of aGVHD cases within cluster 1 was 42% (14/33), compared to 9% (4/45) in cluster 2. A Chi-square test showed a significant difference in aGVHD proportions between the clusters (p = 0.001). Additionally, the severity of aGVHD was higher in cluster 1 compared to cluster 2. Cluster 1 included all the grade IV cases (6/6) and 73% (8/11) of the grade III cases, whereas no patients with grade IV were present in cluster 2 (**Figure 3**). Lastly, a correlation analysis was performed on the eight miRNAs associated with the risk of developing aGVHD (**Figure 4A**). A relatively strong correlation was observed between the expression levels of certain miRNAs. Notably, most miRNAs were upregulated in aGVHD, while two miRNAs, miR-664a-5p and miR-3679-5p, were upregulated in patients who later did not develop aGVHD (**Figure 4A**).

### 3.6 EASIX score is strongly correlated with distinct miRNA

The Endothelial Activation and Stress Index (EASIX) is an emerging biomarker-based tool used to predict outcomes after allo-HSCT, particularly the risk of non-relapse mortality (10, 12). It is calculated using serum levels of lactate dehydrogenase (LDH), creatinine, and platelet count, serving as a reflection of endothelial dysfunction and systemic stress.

**Figure 5:**
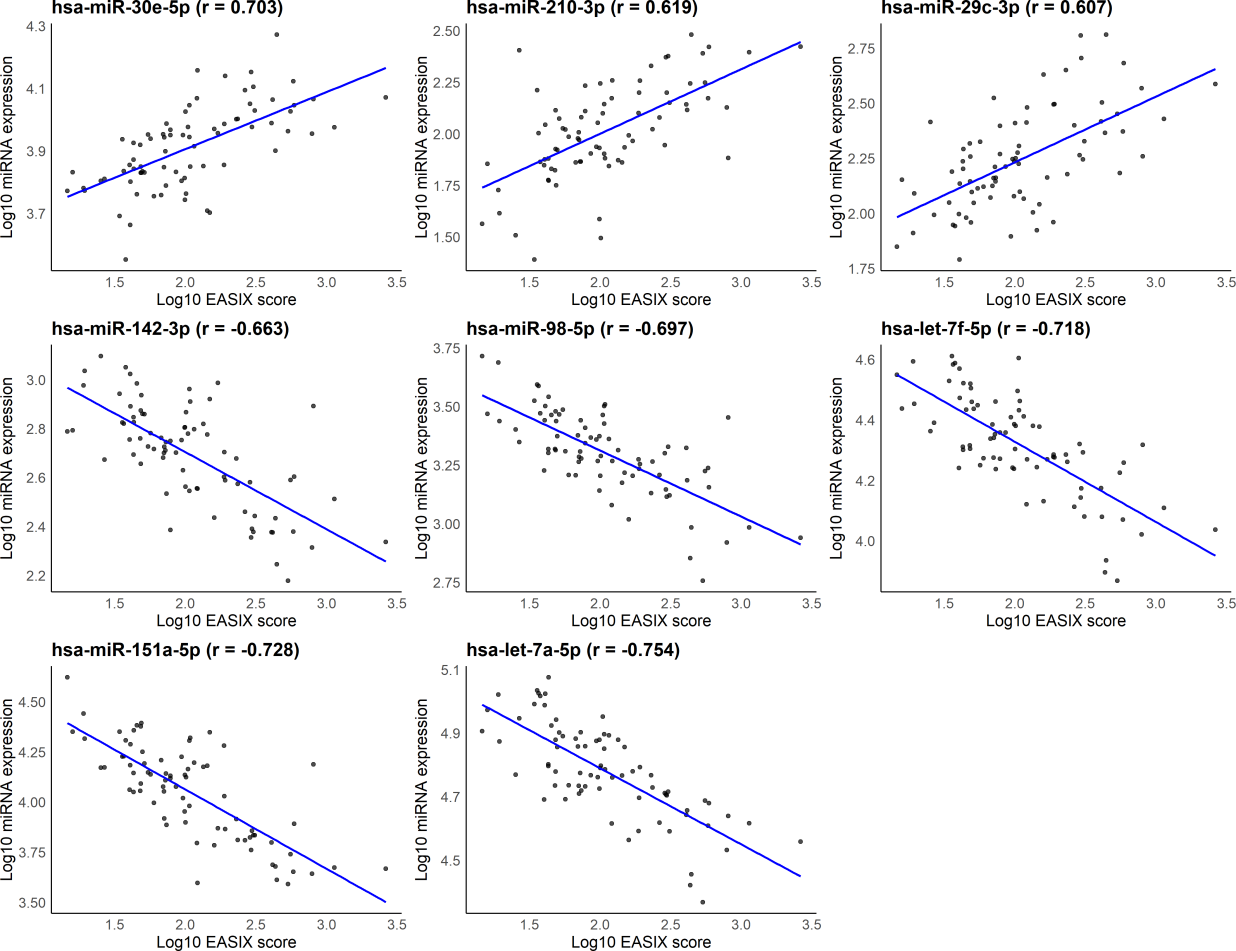
Scatter plots of correlation between EASIX score and miRNA expression. Eight distinct miRNAs that showed either a significant positive or negative correlation with the EASIX score were identified. The different miRNAs, along with their correlation coefficients, are given in the figure.

In this study, we investigated whether the calculated EASIX scores in our patient cohort were associated with the expression patterns of specific miRNAs. Eight distinct miRNAs were identified that showed either a significant positive or negative correlation with the EASIX score, defined as an absolute Spearman correlation > 0.6 and a Benjamini-Hochberg corrected p-value < 0.05 (**Figure 5**). Among these, some were positively correlated, while others were negatively correlated, suggesting potential molecular links between endothelial stress and miRNA regulation. Notably, none of the eight miRNAs identified as correlating with EASIX score overlapped with the eight miRNAs previously identified as predictors of aGVHD (**Figure 4B**).

## 4 Discussion

In this study, we investigated whether pre-transplant circulating miRNA expression profiles could serve as predictive markers for the development of complications after allo-HSCT, especially for aGVHD. Our findings highlight the potential clinical utility of a serum miRNA panel in predicting aGVHD, with potential significant implications for early risk stratification and personalized prophylactic strategies. The identification of miRNA signatures prior to transplantation as predictive of aGVHD is of particular importance in the context of allo-HSCT, where early intervention may significantly alter patient outcomes (4). Most previous studies have evaluated miRNA expression after the onset of GVHD symptoms, thus limiting their predictive capacity (7, 8). In contrast, we demonstrate that specific serum miRNA expression patterns detectable before conditioning therapy and stem cell infusion are associated with later development of clinically significant grade II-IV aGVHD.

Through ROC analysis, we identified eight miRNAs—miR-664a-5p, miR-20b-5p, miR-93-5p, miR-25-3p, miR-1224-5p, miR-106b-5p, miR-454-3p, and miR-3679-5p—that were associated with increased or decreased risk of developing aGVHD. Notably, these miRNAs demonstrated acceptable predictive performance (AUC > 0.7) in the unvalidated model, and the combined eight-miRNA panel yielded an AUC of 0.855. While this value decreased to 0.692 after bootstrap validation, the predictive model remained statistically significant. This decline in AUC post-validation likely reflects overfitting in the initial model and highlights the importance of validation in independent cohorts. Nonetheless, the results are promising and suggest that miRNA-based profiling may offer a valuable tool for aGVHD risk assessment prior to transplantation.

Previous studies have linked multiple microRNAs, e.g., miR-155 and miR-146a, to the pathogenesis of aGVHD after transplantation (13–16). However, the eight miRNAs identified in this study have not previously been linked to aGVHD, and were measured prior to transplantation. A majority of the identified miRNAs were upregulated in patients who later developed aGVHD, which may reflect a pre-existing difference in immune or inflammatory status. This observation is broadly consistent with emerging evidence indicating that pre-transplant immune characteristics, endothelial function, and inflammatory signaling may influence post-transplant outcomes (2, 17–19), although causal inferences cannot be drawn from our present study. Notably, miR-664a-5p and miR-3679-5p showed an inverse association with aGVHD incidence, with higher levels observed in patients who did not develop the condition. While this pattern could be consistent with a potential protective or regulatory role, these findings will require validation in independent cohorts and functional studies.

Hierarchical clustering based on the eight identified miRNAs further supported their clinical relevance. This analysis revealed two distinct patient subgroups, with significantly different incidences and severities of aGVHD. Cluster 1, characterized by higher expression levels of risk-associated miRNAs, contained the vast majority (14/18) of patients who developed aGVHD grade II-IV. Moreover, this cluster included all six grade IV cases and the majority of grade III cases, suggesting that the miRNA profile not only correlates with the occurrence of aGVHD, but may also reflect its eventual severity. This is also in concordance with previous findings (20). In contrast, Cluster 2 included primarily patients without GVHD or with only mild disease. The significant difference in GVHD incidence between clusters (42% vs. 9%, p = 0.001) underscores the potential of miRNA-based clustering to stratify patients by risk and tailor monitoring or prophylactic strategies accordingly. Such pre-transplant stratification could allow for targeted intensification of GVHD prophylaxis in high-risk patients or alternative donor or conditioning choices in borderline cases (21, 22).

Several of the miRNAs identified have been previously implicated in immune regulation, T-cell differentiation, or inflammatory signaling, providing biological plausibility to our findings. For example, miR-20b-5p, miR-93-5p, and miR-106b-5p are members of the miR-106a/363 and miR-106b/25 clusters, which are known to be involved in inflammation and carcinogenesis (23). Dysregulation of these miRNAs has been linked to autoimmunity and inflammatory diseases, suggesting that their elevated levels in patients who later develop aGVHD may reflect a primed or dysregulated immune system prior to transplantation.

The upregulation of miR-25-3p and miR-454-3p has been associated with endothelial activation and apoptosis in prior studies, indicating a possible link between vascular stress and subsequent immune activation in GVHD pathogenesis (24, 25). In contrast, miR-664a-5p and miR-3679-5p, which were elevated in patients who did not develop aGVHD, and miRNAs may have anti-inflammatory or immunological downregulatory roles (26). This dual pattern, pro-inflammatory and potentially protective miRNAs, highlights the complexity of miRNA-mediated regulation and supports the idea that aGVHD development is influenced by the balance of opposing immune forces even before transplant procedures commence.

In addition to exploring miRNAs predictive of aGVHD, we examined their relationship with the EASIX score, a composite marker of endothelial dysfunction and systemic stress derived from LDH, creatinine, and platelet count (10, 12, 27). Our analysis revealed that eight miRNAs—miR-30e-5p, miR-210-3p, miR-29c-3p, miR-142-3p, miR-98-5p, let-7f-5p, miR-151a-5p, and let-7a-5p—were significantly correlated with the EASIX score, some positively, others negatively, indicating that distinct miRNA expression patterns may reflect ongoing endothelial stress and dysfunction (28). The functions of some of these miRNAs have been thoroughly researched, while the functions of others remain unclear. For instance, low levels of miR-142-3p and members of the let-7 family have been associated with poor prognosis and reduced patient survival (29, 30). The present study found similar results, as these miRNAs were inversely correlated with the EASIX score. Consequently, lower miRNA expression levels were associated with higher EASIX scores, and thus indirectly with poorer prognosis.

Importantly, none of the EASIX-associated miRNAs overlapped with those predictive of aGVHD, suggesting that the biological pathways underlying systemic endothelial dysfunction and immune-mediated GVHD risk may be partially independent. This divergence underscores the multifactorial nature of allo-HSCT outcomes, where both endothelial integrity and immune priming play crucial roles (31). The pathogenesis of aGVHD is multifactorial, and the significance of the hematopoietic stem cell graft composition of CD34^+^ progenitor cells and different immune cell subsets remains contradictory (32). In a previous study, we identified no correlations between the transplanted doses of hematopoietic stem cells or T cells and the incidence of aGVHD post-transplant (33). The present study confirms these findings, as no significant differences in CD34+ cell dose, CD3+ cell dose, or donor characteristics were observed between patients who developed aGVHD grade II–IV and those who did not. This further supports the notion that graft composition alone does not predict the development of aGVHD.

The identification of pre-transplant serum miRNA signatures predictive of aGVHD could open new avenues for personalized medicine in allo-HSCT. With appropriate validation, these miRNA panels could be developed into routine blood tests to identify high-risk patients before the onset of clinical symptoms or tissue damage. Such predictive testing could guide decisions regarding GVHD prophylaxis intensity, donor selection, conditioning regimen, and post-transplant monitoring. Ultimately, such early risk stratification could enable timely intervention and reduce the incidence and severity of aGVHD.

However, several limitations must be acknowledged. The study cohort, although well-characterized, remains relatively small (n = 78), and the findings require validation in independent, larger, and more diverse populations. Additionally, the low negative predictive values observed in bootstrap validation suggest that while the miRNA signature is highly specific, it may not be sufficiently sensitive to serve as a standalone screening tool. Limitations are also related to the exploratory design and multiple testing, increasing the risk of model overfitting and potentially reducing clinical applicability. Notably, it should be noticed NPV of the combined model is modest (0.447), indicating limited sensitivity and a reduced ability to reliably exclude the risk of aGVHD when the test is negative. This substantially limits the utility of the miRNA panel as a standalone screening tool in a clinical setting, where high NPV is typically required. These findings suggest that the predictive value of the miRNA signature alone is insufficient. A more promising approach may involve integrating the miRNA panel with established clinical and biological parameters, such as EASIX (34), inflammatory cytokines (35), or genetic markers (36), which could enhance overall discrimination and risk stratification (4). From a mechanistic standpoint, further studies are needed to elucidate the functional roles of the identified miRNAs in GVHD pathophysiology. Experimental validation using *in vitro* models of T-cell activation and endothelial injury, or *in vivo* transplant models, could help clarify whether these miRNAs are merely biomarkers or active participants in disease development.

Another limitation is related to the heterogeneity of the patient cohort with respect to demographic data, underlying disease and conditioning regimens (**Table 1**), which may influence aGVHD risk and potentially confound observed associations. Due to the limited sample size and number of outcome events, multivariable regression analyses to adjust for these established clinical risk factors were not considered relevant and feasible. As a result, residual confounding cannot be excluded, and the reported associations between miRNA expression and aGVHD should be interpreted with caution. Furthermore, although eight miRNAs were significantly correlated with the EASIX score, none showed a statistically significant association with overall survival or non-relapse mortality in subsequent survival analyses, which may be attributable to the limited sample size and the relatively low number of events. Finally, it should be emphasized that this study has a mainly exploratory and hypothesis-driven nature, and that its clinical implications, including integration into pre-transplant assessment, need further preclinical and clinical evaluation.

## 5 Conclusion

In summary, this study demonstrates that serum miRNA profiles obtained prior to allo-HSCT can predict the risk and severity of subsequent aGVHD. The eight-miRNA signature identified here holds promise as a tool for early patient stratification and risk-guided intervention. Moreover, the distinct set of miRNAs associated with EASIX underscores the value of integrating endothelial and immune biomarkers to capture the multifaceted biology of post-transplant complications. Future validation and mechanistic studies will be crucial for translating these findings into clinical practice and enhancing allo-HSCT outcomes.

## Data Availability

The raw data supporting the conclusions of this article will be made available by the authors, without undue reservation.
